# Sex-specific maternofetal innate immune responses triggered by group B *Streptococci*

**DOI:** 10.1038/s41598-019-45029-x

**Published:** 2019-06-13

**Authors:** Marie-Julie Allard, Antoine Giraud, Mariela Segura, Guillaume Sebire

**Affiliations:** 1 Department of Pediatrics, McGill University, Montreal, QC Canada; 2 EA 4607 SNA EPIS, University of Lyon, Jean Monnet University, Saint-Etienne, France; 3 Neonatal Intensive Care Unit, Department of Pediatrics, Saint-Etienne University Hospital, Saint-Etienne, France; 4 Department of Infectiology and Microbiology, Faculty of Veterinary Medicine, Université de Montréal, Saint-Hyacinthe, QC Canada; 5 Department of Pediatrics, Université de Sherbrooke, Sherbrooke, QC Canada

**Keywords:** Interleukins, Intrauterine growth, Bacterial infection, Acute inflammation, Experimental models of disease

## Abstract

Group B *Streptococcus* (GBS) is one of the most common bacteria isolated in human chorioamnionitis, which is a major risk factor for premature birth and brain injuries. Males are at greater risk than females for developing lifelong neurobehavioural disorders, although the origins of this sex bias remain poorly understood. We previously showed that end-gestational inflammation triggered by GBS led to early neurodevelopmental impairments mainly in the male rat progeny. Identifying key inflammatory players involved in maternofetal immune activation by specific pathogens is critical to develop appropriate novel therapeutic interventions. We aimed to map out the GBS-induced profile of innate immune biomarkers in the maternal-placental-fetal axis, and to compare this immune profile between male and female tissues. We describe here that the GBS-induced immune signalling involved significantly higher levels of interleukin (IL)-1β, cytokine-induced neutrophil chemoattractant-1 (CINC-1/CXCL1) and polymorphonuclear cells (PMNs) infiltration in male compared to female maternofetal tissues. Although male – but not female – fetuses presented increased levels of IL-1β, fetuses from both sexes *in-utero* exposed to GBS had increased levels of TNF-α in their circulation. Levels of IL-1β detected in fetal sera correlated positively with the levels found in maternal circulation. Here, we report for the first time that the maternofetal innate immune signalling induced by GBS presents a sexually dichotomous profile, with more prominent inflammation in males than females. These sex-specific placental and fetal pro-inflammatory responses are in keeping with the higher susceptibility of the male population for preterm birth, brain injuries and neurodevelopmental disorders such as cerebral palsy and autism spectrum disorders.

## Introduction

One of the most common bacteria causing chorioamnionitis is group B *Streptococcus* (GBS; *Streptococcus agalactiae*), which is isolated in about 15% of cases^1–4^. Serotype Ia is the most prevalent GBS serotype infecting maternal or fetal tissues^5,6^. GBS stimulates the innate immune responses by its pathogen-associated molecular patterns engaging different toll-like receptor (TLR)s, including TLR2/6, and by its β-hemolysin component acting on the inflammasome NOD-like receptor (NLR)-P3 pathway^7–14^. Activation of TLRs and NLRP3 pathways stimulate the synthesis of the pro-inflammatory cytokines interleukin (IL)-1β, tumor necrosis factor (TNF)-α, IL-6 and IL-18 in macrophages, dendritic cells and polymorphonuclear cells (PMNs)^14,15^. In the context of chorioamnionitis, these cytokines induce the release of chemokines (C-X-C), which attract and activate predominantly PMNs that produce prostaglandin and matrix metalloproteinases (MMPs)^14,15^. High levels of prostaglandin and MMPs can trigger amniotic membrane rupture and uterine contractions leading to preterm delivery^14,15^. PMNs can amplify their own recruitment via the production of IL-1β at infection sites^12^. In addition, PMNs play a critical role in controlling GBS invasion through the formation of deoxyribonucleic acid (DNA) PMN extracellular traps, enabling PMNs to catch and kill bacteria^12,16,17^. PMN-derived DNA is associated with antimicrobial proteins including the calprotectin heterodimer (S100A8/S100A9), which is found in higher proportion in preterm pre-labour rupture of membranes^18–22^.

Clinical and preclinical evidence show that perinatal activation of the innate immune system involving an altered profile of released cytokines correlates with the level of severity of neurological sequelae in the progeny, such as cerebral palsy, autism spectrum disorder, and attention-deficit/hyperactivity disorder (ADHD)^23–26^. All these perinatal neurobehavioural morbidities affect collectively 1 out of 6 children^27^. They are characterized by a skewed *sex ratio* toward males, ranging from 1.4:1 to 3:1^28–31^. This ratio is not solely explained by the effect of X-linked genes, but appears to result from a sex dichotomous environmental impact on the genetic backbone^28–31^.

Our lab has developed rat models of GBS-induced chorioamnionitis. We previously showed that male rat offspring presented autistic-like traits, in sharp contrast with hyperactive behaviour in females^32–34^. The GBS-exposed offspring presented dysmyelinated forebrain white matter reminiscent of those observed in human perinatal injuries^32–34^. In this same model, end-gestational GBS-induced acute chorioamnionitis was characterised by a more prominent PMN infiltration in placentas of males compared to female offspring, raising the hypothesis of a relationship between the sex-biased inflammatory response and the distinct neurobehavioural phenotypes^32^. In this work, we used our model to determine whether GBS-induced chorioamnionitis induces a sexually dichotomous innate inflammatory process driven by IL-1β, IL-1-induced chemokine cytokine-induced neutrophil chemoattractant-1 (CINC-1/CXCL1) attracting PMNs, and PMN-produced S100A9 and MMP-8.

## Results

### Systemic GBS inoculation in dams led to placental infection

**Figure 1:**
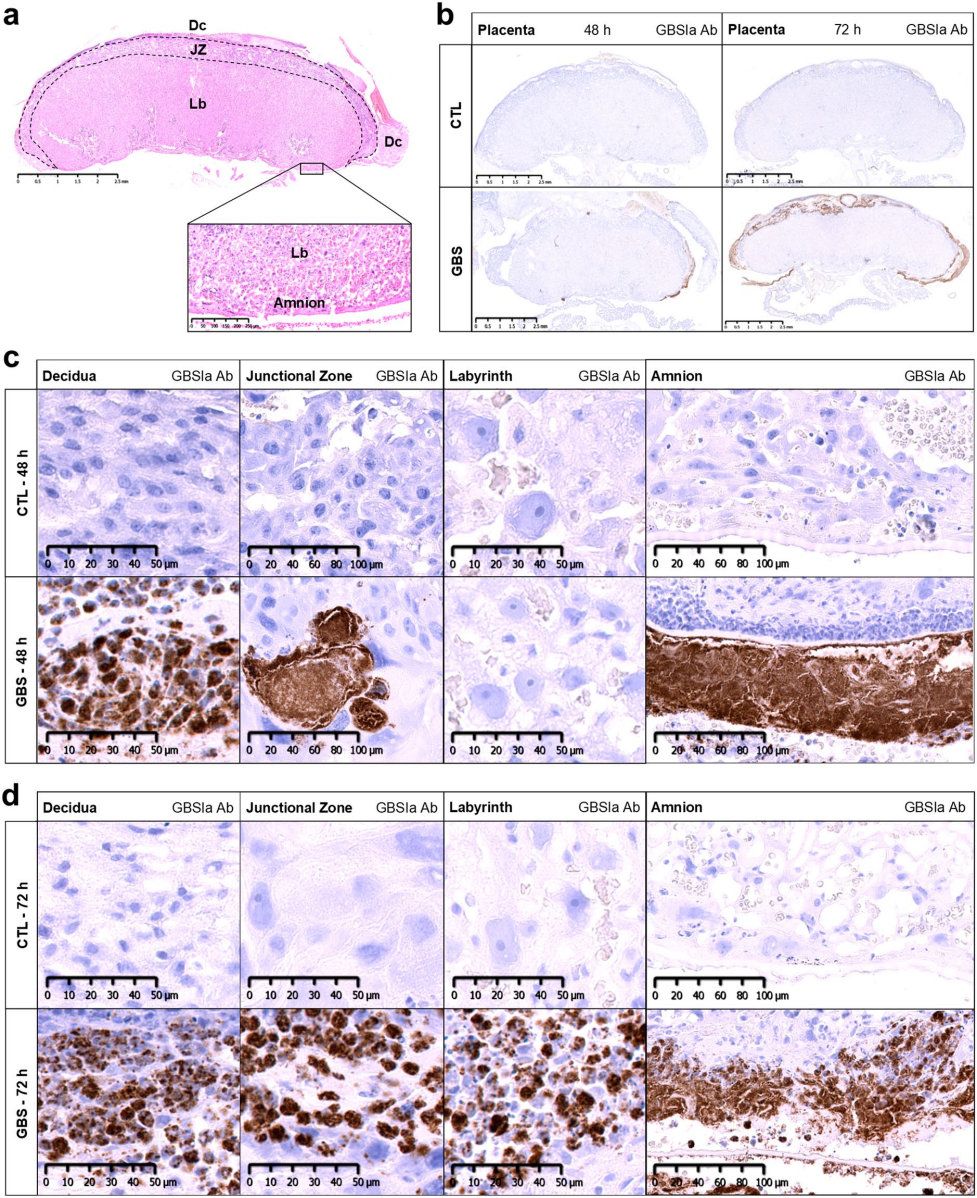
Placental group B *Streptococcus* (GBS) infection. (**a**) Global anatomical aspect of coronal section of the placenta stained by hematoxylin and eosin presenting the maternal (decidua [Dc]), maternofetal (junctional zone [JZ]) and fetal (labyrinth [Lb]) compartments, as well as the amnion. (**b**) Representative images of immunohistochemical detection of GBS serotype Ia showing placentas of dams exposed or unexposed (CTL) to GBS. Scale bar, 2.5 mm. (**c**,**d**) Representative GBS-stained bacteria infiltrates in the decidua, junctional zone, labyrinth and amnion at 48 h (**c**) and 72 h (**d**) post-inoculation by GBS. Abbreviations: Ab, Antibody; CTL, Control; Dc, Decidua; GBS, Group B *Streptococcus*; h, hour; JZ, Junctional Zone; Lb, Labyrinth.

We used our established rat model of live GBS-induced acute chorioamnionitis leading to sex-specific neurobehavioural impairments^32^. Briefly, pregnant Lewis rats were inoculated intraperitoneally with 10^8^ CFU of β-hemolytic GBS serotype Ia or sterile saline 0.9% (control; CTL) at gestational day (G) 19. *In-situ* and ELISA analyses were performed at 48 h (G21) and 72 h (G22) post-injection. At 48 h post-infection, massive GBS infiltrates were detected in the amnion, decidua and junctional zone (to a lesser extent) of GBS-exposed placentas as seen by immunohistochemistry (Fig. 1a–c). No infiltration was seen in the labyrinth layer, however, at 72 h post-infection, GBS infiltrates were detected in this area (Fig. 1b,d). In contrast, mock-infected rats (saline controls) did not show any GBS infiltrates at 48 h and 72 h (Fig. 1c,d). No difference in the extent of GBS infiltration was observed between litter-matched male and female placentas. No mortality in dams nor delivery before Caesarian (C)-sections occurred in either GBS-exposed or unexposed groups.

### Higher PMN chemoattraction, infiltration, and release of PMN-produced proteins in male than female placentas infected by GBS

**Figure 2:**
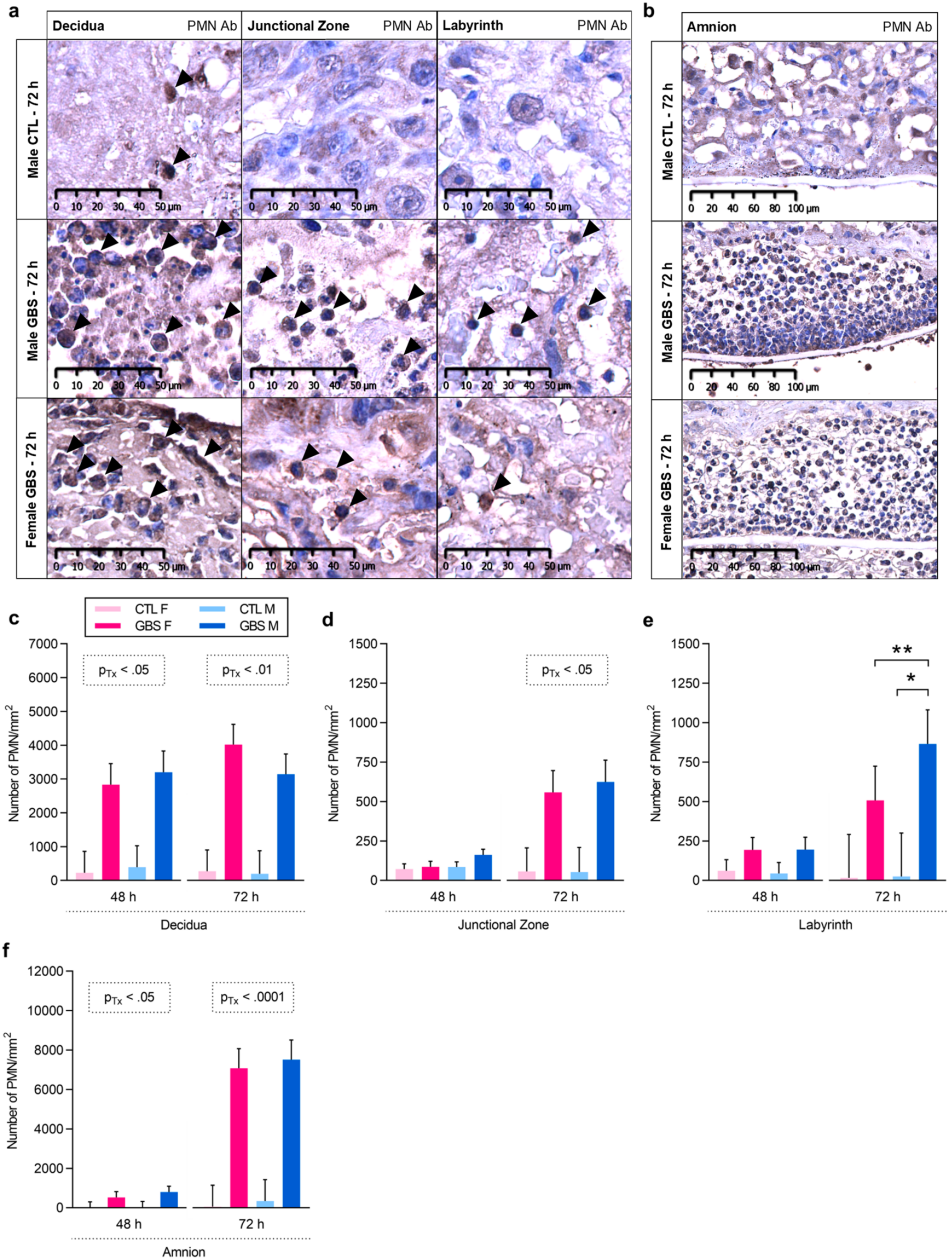
Prenatal GBS infection induces sexually dichotomous polymorphonuclear cells (PMNs) infiltration in placentas. (**a**,**b**) Representative images of PMN infiltrates in the decidua, junctional zone, labyrinth (**a**) and amnion (**b**) in placentas at 72 h post-injection. (**c**–**f**) Mean density of PMNs detected by immunohistochemistry in the decidua (**c**), junctional zone (**d**), labyrinth (**e**) and amnion (**f**) at 48 and 72 h post-injection. Analyses were done by linear mixed model and significant results are shown in the dashed box. Sidak’s multiple comparisons were used when the interaction between sex and treatment was significant. *P < 0.05, **P < 0.01. Bars represent mean ± SEM. Number (n) of placentas: n = 3 [48 h] and n = 6 [72 h] per sex in the GBS group, and n = 5 [48 h] and n = 5 [72 h] per sex in the CTL group. One male and one female placentas were used per litter. Abbreviations: Ab, Antibody; CTL, Control; F, Female; GBS, Group B *Streptococcus*; h, hour; M, Male; Tx, Treatment.

We first measured PMN densities in the decidua, junctional zone, labyrinth and amnion. At 48 h, GBS-infected placentas from both sexes displayed increased PMN density in the decidua and amnion – but not in the junctional zone or labyrinth – compared to saline-exposed placentas (Fig. 2a–f). At 72 h, increased PMN densities were detected in the decidua, amnion and junctional zone of GBS-infected placentas compared to controls (Fig. 2a–d,f). In the labyrinth layer, there was a significant interaction between sex and treatment for the density of PMN: GBS-infected male placentas presented an increased PMN density compared to same-sex controls and a 3.0-fold increase compared to litter-matched GBS-exposed females at 72 h (Fig. 2a,e). At both 48 and 72 h post-infection, massive PMN infiltrates were observed in the amnion adjacent to the labyrinth, corresponding to the same zone in which a large amount of GBS was seen in (Figs 1c and 2b).

**Figure 3:**
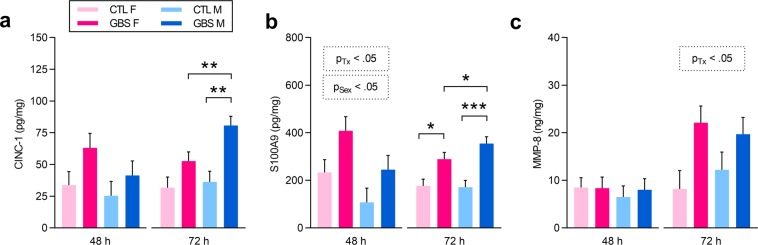
GBS-induced acute chorioamnionitis is associated with sex-specific placental levels of CINC-1 and S100A9 and increased MMP-8 production. (**a**–**c**) Mean placental concentrations of CINC-1/CXCL1 (**a**), S100A9 (**b**) and MMP-8 (**c**) detected by ELISA at 48 and 72 h post-injection. Analyses were done by linear mixed model and significant results are shown in the dashed box. Sidak’s multiple comparisons were used when the interaction between sex and treatment was significant. *P < 0.05, **P < 0.01, ***P < 0.001. Bars represent mean ± SEM. Number (n) of placentas: n = 4 [48 h] and n = 6 [72 h] per sex in the GBS group, and n = 5 [48 h] and n = 5 [72 h] per sex in the CTL group. One male and one female placentas were used per litter. Abbreviations: CTL, Control; CINC-1, Cytokine-Induced Neutrophil Chemoattractant-1; F, Female; GBS, Group B *Streptococcus*; h, hour; M, Male; MMP, Matrix Metalloproteinase; Tx, Treatment.

To investigate whether there were sex differences in PMN-attractant and PMN-produced biomarkers, we quantified CINC-1 (corresponding to CXCL1) and the alarmin S100A9 and MMP-8 in placentas by ELISA. A significant interaction between sex and treatment for CINC-1 concentrations was revealed at 72 h: GBS-infected male placentas displayed increased CINC-1 titers compared to same-sex controls, and a 1.6-fold increase compared to litter-matched females (Fig. 3a). At 48 h, GBS-exposed placentas from both sexes contained increased amounts of S100A9 proteins compared to controls (Fig. 3b). Placentas associated with females from both experimental conditions presented increased titers of S100A9 compared to males (Fig. 3b). At 72 h, a significant interaction between sex and treatment was revealed for S100A9 levels: increased titers of S100A9 were detected in GBS-infected placentas from both sexes compared to controls, and GBS-exposed male placentas had a 1.4-fold increased concentration of S100A9 compared to litter-matched females (Fig. 3b). We then measured levels of MMP-8, released mainly from PMNs, in placentas as a marker of PMN activation^35,36^. At 72 h, elevated levels of MMP-8 were detected in GBS-infected placentas from both sexes, compared to controls (Fig. 3c), with no sex-effect observed.

### Expression of placental pro-inflammatory and anti-inflammatory mediators was sex-specific in GBS-infected placentas

**Figure 4:**
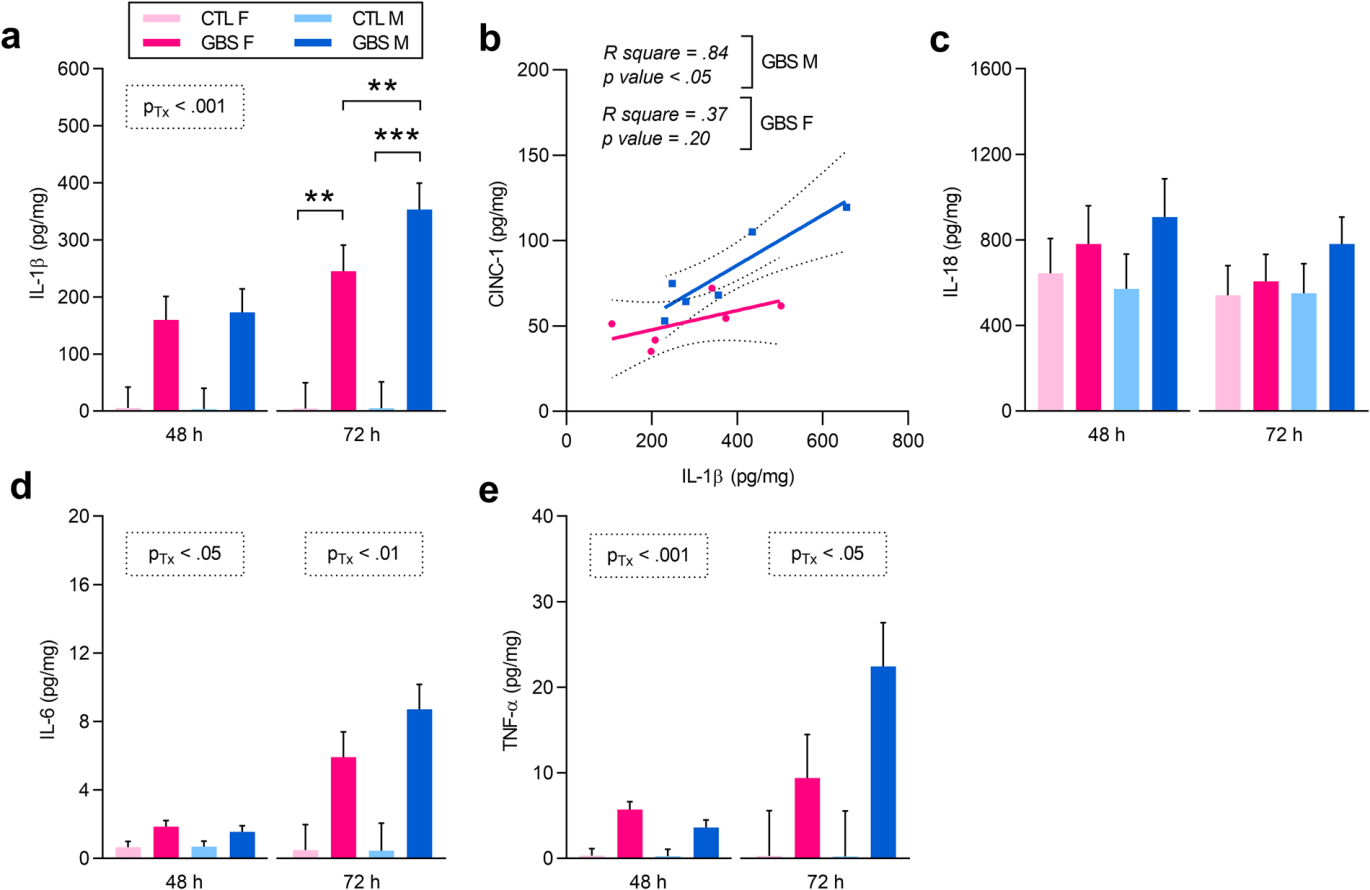
GBS-infected placentas displayed a sex dichotomous pro-inflammatory profile. (**a**) Mean concentration of IL-1β in placentas at 48 and 72 h quantified by ELISA. Comparisons between GBS-infected *versus* CTL placentas by linear mixed model, with Sidak’s multiple comparisons when the interaction between sex and treatment was significant. **P < 0.01, ***P < 0.001. (**b**) Correlation between levels of IL-1β and CINC-1/CXCL1 at 72 h post-injection. The within-subject correlation between IL-1β and CINC-1 levels was analysed by linear regression. (**c**–**e**) Mean levels of IL-18 (**c**), IL-6 (**d**) and TNF-α (**e**) detected in placentas at 48 and 72 h post-injection by ELISA. Analyses were done by linear mixed model and significant results are shown in the dashed box. Bars represent mean ± SEM. Number (n) of placentas: n = 4 [48 h] and n = 6 [72 h] per sex in the GBS group, and n = 5 [48 h] and n = 5 [72 h] per sex in the CTL group. One male and one female placentas were used per litter. Abbreviations: CTL, Control; CINC-1, Cytokine-Induced Neutrophil Chemoattractant 1; F, Female; GBS, Group B *Streptococcus*; h, hour; IL, Interleukin; M, Male; TNF-α, Tumor Necrosis Factor-α; Tx, Treatment.

We initially measured placental IL-1β by ELISA, as this pro-inflammatory cytokine has been shown to play a crucial role in the production of the PMN chemoattractant CINC-1/CXCL1 in GBS models^14,37^. At 48 h, GBS-infected placentas from both sexes displayed elevated levels of IL-1β compared to controls (Fig. 4a). At 72 h, a significant interaction between sex and treatment was revealed: increased titers of IL-1β were detected in GBS-infected placentas from both sexes compared to same-sex controls, and GBS-exposed males had a 1.5-fold increase compared to litter-matched females (Fig. 4a). These levels of IL-1β correlated positively with the production of CINC-1 in GBS-infected male – but not female – placentas (Fig. 4b).

We then studied other pro-inflammatory cytokines that are involved in GBS infection and chorioamnionitis, namely IL-18, IL-6 and TNF-α, to evaluate whether they presented the same sexually dichotomous profile. No difference was revealed for IL-18 levels between GBS-exposed and unexposed placentas (Fig. 4c). At 48 and 72 h, increased titers of IL-6 and TNF-α were detected in GBS-infected placentas from both sexes compared to controls (Fig. 4d,e), but no sex-effect was observed.

**Figure 5:**
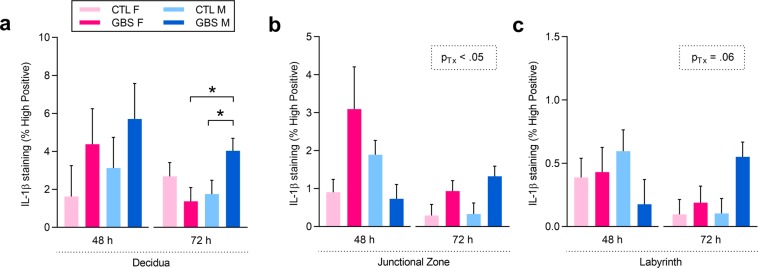
*In-situ* immunohistochemical analysis revealed sex- and placental compartment-specific increases of IL-1β following GBS-induced acute chorioamnionitis. (**a**–**c**) Percentage (%) of high positive staining for IL-1β in the decidua (**a**), junctional zone (**b**) and labyrinth (**c**) placental compartments at 48 and 72 h post-injection. Analyses were done by linear mixed model and significant results are shown in the dashed box. Sidak’s multiple comparisons were used when the interaction between sex and treatment was significant. *P < 0.05. Bars represent mean ± SEM. Number (n) of placentas: n = 3 [48 h] and n = 6 [72 h] per sex in the GBS group, and n = 5 [48 h] and n = 5 [72 h] per sex in the CTL group. One male and one female placentas were used per litter. Abbreviations: CTL, Control; F, Female; GBS, Group B *Streptococcus*; h, hour; IL, Interleukin; M, Male; Tx, Treatment.

To determine whether the sexually dichotomous effect in IL-1β production was specific to a placental compartment, we measured *in-situ* the IL-1β staining intensity in the decidua, junctional zone and labyrinth. At 72 h, a significant interaction between sex and treatment was revealed in the decidual compartment: GBS-infected male placentas displayed increased IL-1β staining intensity compared to same-sex controls and a 4.9-fold increased intensity compared to litter-matched females (Fig. 5a). At 72 h, GBS-infected placentas from both sexes displayed increased IL-1β staining intensity in the junctional zone and a trend towards an increase in the labyrinth layer – compared to controls (Fig. 5b,c).

**Figure 6:**
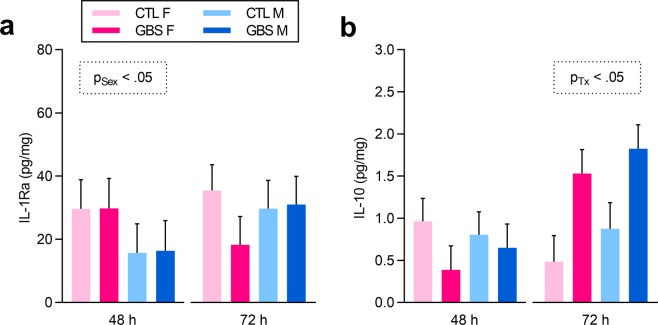
GBS-infected placentas displayed a sexually dichotomous anti-inflammatory profile. (**a**,**b**) Mean protein levels of IL-1Ra (**a**) and IL-10 (**b**) detected in placentas at 48 and 72 h post-injection by ELISA. Analyses were done by linear mixed model and significant results are shown in the dashed box. Number (n) of placentas: n = 3 [48 h] and n = 6 [72 h] per sex in the GBS group, and n = 5 [48 h] and n = 5 [72 h] per sex in the CTL group. One male and one female placentas were used per litter. Abbreviations: CTL, Control; F, Female; GBS, Group B *Streptococcus*; h, hour; IL, Interleukin; IL-1Ra, Interleukin-1 Receptor antagonist; M, Male; Tx, Treatment.

Levels of endogenous IL-1 receptor antagonist (IL-1Ra) – which binds to the IL-1R to block the IL-1β-induced proinflammatory signalling cascade – and the anti-inflammatory cytokine IL-10 were measured in placentas to assess potential sex biases in anti-inflammatory responses. At 48 h, females presented higher basal IL-1Ra titers than male placentas, and these levels did not significantly change due to GBS infection (Fig. 6a). At 72 h, increased concentration of IL-10 was detected in GBS-infected placentas from both sexes compared to controls, but no sex effect was observed (Fig. 6b).

### Sexually dichotomous pro-inflammatory responses in fetal circulation associated with GBS-induced acute chorioamnionitis

**Figure 7:**
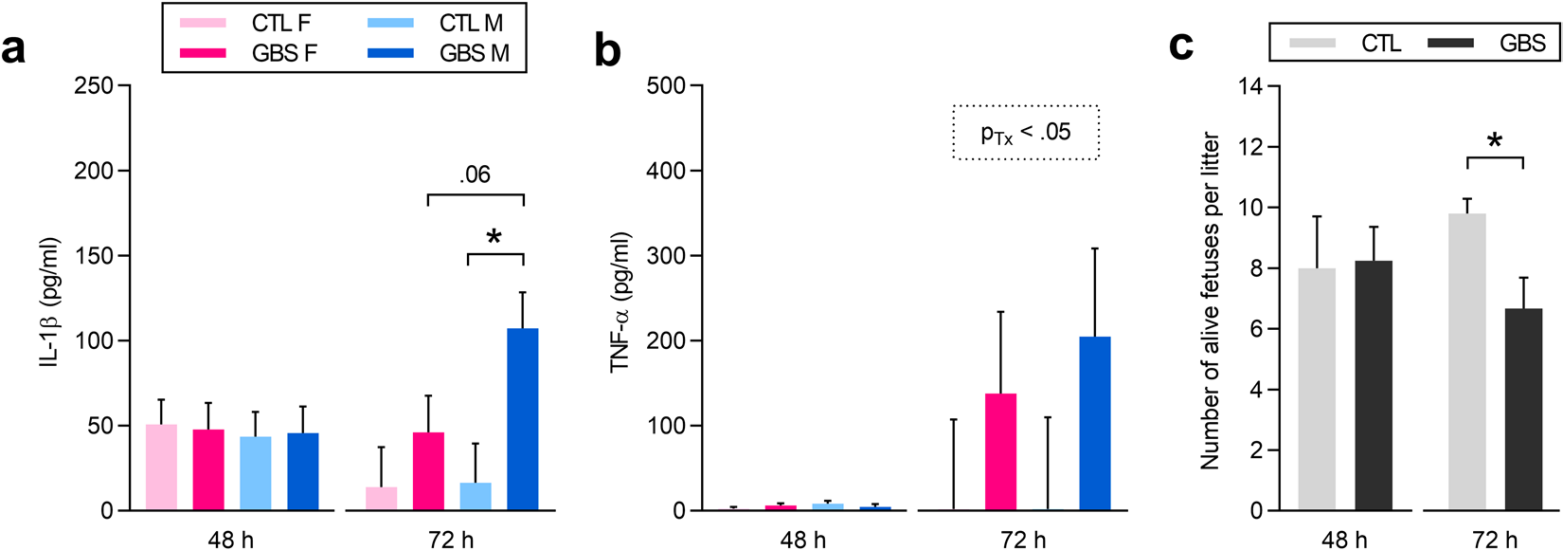
Sex-specific pro-inflammatory responses in the fetal circulation. (**a**,**b**) Mean titers of IL-1β (**a**) and TNF-α (**b**) detected in the fetal sera at 48 and 72 h post-injection. Comparisons between GBS-exposed *versus* CTL fetuses by linear mixed model, with Sidak’s multiple comparisons when the interaction between sex and treatment was significant. *P < 0.05. Bars represent mean ± SEM. Significant results are shown in the dashed box. Number (n) of fetuses for IL-1β: n = 4 [48 h] and n = 6 [72 h] per sex in the GBS group, and n = 4 [48 h] and n = 5 [72 h] per sex in the CTL group. Number (n) of fetuses for TNF-α: n = 4 [48 h] and n = 6 [72 h] per sex in the GBS group, and n = 4 [48 h] and n = 5 [72 h] per sex in the CTL group. **c** Mean number of alive fetuses per litter at 48 and 72 h post-injection. *P < 0.05. Bars represent mean ± SEM. Number (n) of litters: n = 4 [48 h] and n = 6 [72 h] per sex in the GBS group, and n = 5 [48 h] and n = 5 [72 h] per sex in the CTL group. Comparisons between GBS-exposed *versus* CTL litters by two-tailed Student’s T tests. Abbreviations: CTL, Control; F, Female; GBS, Group B *Streptococcus*; h, hour; IL, Interleukin; M, Male; TNF-α, Tumor Necrosis Factor-α; Tx, Treatment.

We then measured relevant pro-inflammatory cytokines in the fetal sera – namely IL-1β, TNF-α and IL-6 – to evaluate whether sex differences detected in placentas were present in the associated fetuses. At 72 h, there was a significant interaction between sex and treatment for IL-1β titers in fetal sera: GBS-exposed male – but not female – fetuses presented increased titers of IL-1β compared to same-sex controls (Fig. 7a), and there was a trend toward a 3.2-fold increase in levels of IL-1β compared to GBS-exposed females (Fig. 7a). Fetuses from both sexes *in-utero* exposed to GBS at 72 h had increased levels of TNF-α in their circulation compared to controls (Fig. 7b). There was no difference for IL-6 levels between experimental conditions in the fetal sera (data not shown).

There were fewer alive fetuses at 72 h post-treatment in GBS-exposed litters compared to controls (Fig. 7c). No difference in the *sex ratio* of alive fetuses was observed between experimental conditions (data not shown).

### Decreased maternal weight gain and maternal pro-inflammatory environment associated with placental GBS infection

**Figure 8:**
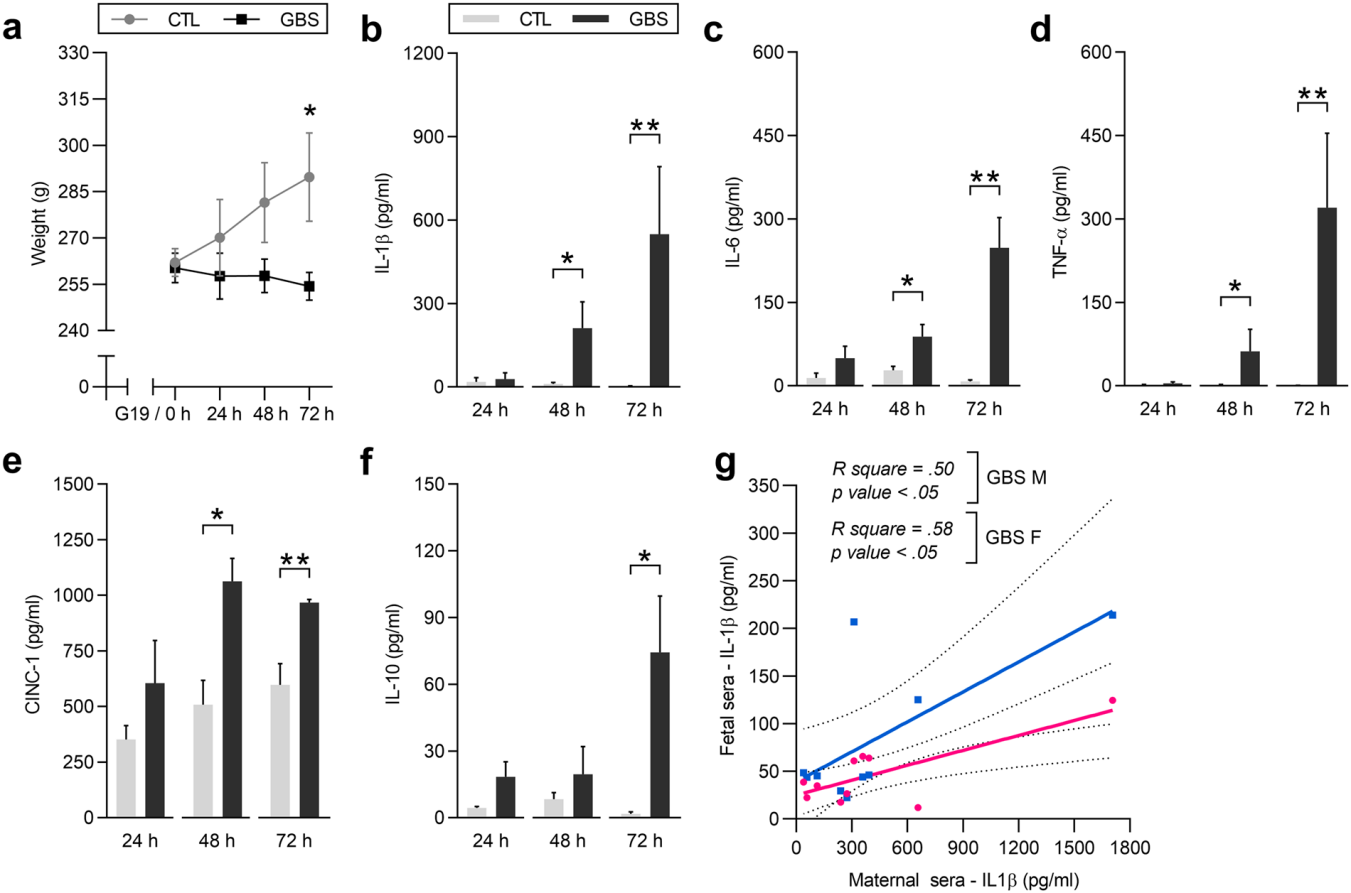
Decreased maternal weight gain and increased pro-inflammatory responses in the maternal circulation associated with GBS-induced acute chorioamnionitis. (**a**) Daily mean maternal weight from G19/0 h to 72 h post-injection. Comparisons between GBS-exposed *versus* CTL dams by repeated measures ANOVA, with Sidak’s multiple comparisons when the interaction between G and treatment was significant. *P < 0.05. Bars represent mean ± SEM. (**b**–**f**) Mean concentrations of IL-1β (**b**), IL-6 (**c**), TNF-α (**d**), CINC-1 (**e**) and IL-10 (**f**) in the maternal sera at 24, 48 and 72 h post-injection. Data sets were analysed by two-tailed unpaired Student’s T test and two-tailed unpaired Mann-Whitney test when data were, respectively, normally and not normally distributed (Shapiro-Wilk test). *P < 0.05, **P < 0.01. Bars represent mean ± SEM. Number (n) of dams: n = 4 [24 h], n = 4 [48 h] and n = 6 [72 h] per sex in the GBS group, and n = 3 [24 h], n = 5 [48 h] and n = 5 [72 h] per sex in the CTL group. (**g**) Correlation between the levels of IL-1β detected in the fetal and maternal sera at 72 h post-injection. The within-litter correlation between IL-1β levels was analysed by linear regression. Abbreviations: CTL, Control; CINC-1, Cytokine-Induced Neutrophil Chemoattractant-1; F, Female; GBS, Group B *Streptococcus*; h, hour; IL, Interleukin; M, Male; TNF-α, Tumor Necrosis Factor-α; Tx, Treatment.

Dams infected by GBS gained less weight than controls, although no fever nor sickness-like behaviour was noticed in any group (Fig. 8a). In the clinical setting, there is an absence of reliable prenatal diagnostic biomarkers of chorioamnionitis. We, therefore, investigated potential inflammatory markers associated with GBS-induced acute chorioamnionitis in maternal blood at 24, 48 and 72 h. At 24 h, no differences were detected for IL-1β, IL-6, TNF-α, CINC-1 and IL-10 between experimental conditions (Fig. 8b–f). At 48 h, increased titers of the pro-inflammatory cytokines IL-1β (20-fold), IL-6 (3-fold), TNF-α (42-fold) and the chemokine CINC-1 (2-fold) were detected in the maternal sera of GBS-infected dams compared to controls (Fig. 8b–e). At 72 h, these differences were more pronounced: pro-inflammatory cytokines IL-1β (313-fold), IL-6 (31-fold) and TNF-α (412-fold), chemokine CINC-1 (1.4-fold). We also detected the anti-inflammatory cytokine IL-10, which was increased (37-fold) in GBS-infected dams compared to controls (Fig. 8b–f). S100A9 protein was not detected in the maternal circulation of both GBS-exposed and unexposed dams (data not shown). At 72 h, the levels of IL-1β in fetal sera correlated positively with the levels of IL-1β in litter-matched maternal sera (Fig. 8g).

## Discussion

We used our established GBS experimental model to determine if there was a sex-specific innate immune response *in-utero*. Our results show for the first time a sexually dichotomous innate immune signalling more prominent in males than females in maternofetal tissues following an end-gestational infection. Here we provide novel findings that can be summarized as follows: (*i*) The PMN density was markedly increased in the labyrinth layer of male compared to female placentas infected by GBS serotype Ia; (*ii*) IL-1β, CINC-1/CXCL1 and S100A9 responses were more prominent in male *versus* female placentas infected by GBS; and (*iii*) in contrast to IL-1β, no sex difference was detected for IL-6, TNF-α, and MMP-8 although their levels were increased in GBS-infected placentas. The exaggerated male innate immune response might be related to sex-specific anti-inflammatory responses. In line with this hypothesis, endogenous IL-1Ra was constitutively lower in male *versus* female placentas. Finally, the known neurotoxic cytokine IL-1β displayed a sexually dichotomous increase in the offspring circulation, with higher levels in male – but not female – fetuses, correlating with IL-1β levels in the maternal circulation. This correlation might provide a currently missing and non-invasive biomarker of chorioamnionitis (most often subclinical) allowing the detection and measuring the intensity of fetal inflammation.

The defining feature of acute chorioamnionitis in humans is diffuse infiltration of PMNs^38^. A study conducted in a GBS-induced infection in mice demonstrated that 82% and 7% of IL-1β-positive cells were PMNs and macrophages, respectively, suggesting a crucial role of PMNs in the production of IL-1β and thereby amplifying their own recruitment^12^. IL-1β is a key player in the placental immune response against GBS infection and PMN infiltration, according to preclinical studies of systemic infection conducted in adults^12,39^. In human, increased levels of IL-1β in maternal and fetal serum from mothers colonised by GBS in the urogenital tract, have been associated with preterm deliveries^40^. Elevated IL-1β and lower TNF-α and IL-6 responses were documented following the exposure of *ex-vivo* human choriodecidual tissues to live GBS^41^. These results, in line with ours, suggest a key role of IL-1β in GBS-induced acute chorioamnionitis associated with preterm deliveries. In a preclinical model using adult IL-1β-deficient mice, it was demonstrated that IL-1β had a direct role in triggering the production of the chemokine CINC-1/CXCL1 driving PMN infiltration in GBS-infected organs^14^. Hence, the elevated levels of IL-1β and CINC-1/CXCL1 in GBS-exposed male placentas support the implication of IL-1β in their sex-specific PMN infiltration. In addition, PMNs have been shown to contribute to their own recruitment by producing IL-1β in an infectious context^12^. In human, high expression of PMN-produced S100A9 was detected in chorioamnionitis and was associated with preterm labour without premature membrane rupture^19,42^. S100A9, which is constitutively expressed in PMNs, is also a recognised alarmin that promotes the inflammatory response by increasing the production of CINC-1/CXCL1 and thereby initiating and amplifying PMN chemotaxis^43–46^. The higher concentrations of S100A9 detected in GBS-infected male compared to female placentas might contribute to the skewed *sex ratio* towards males in preterm birth. Our results are in line with a recent clinical study investigating placentas of human twins, in which males have been found to have increased placental inflammation and lesions compared to females^47^.

The perinatal period is critical for brain development. Dysregulation of maternal cytokine levels during gestation has been associated with disturbances of motor and cognitive development, leading, for instance, to cerebral palsy, learning impairments, or autism spectrum disorders^48,49^. We previously showed using the same model that *in-utero* GBS-exposed rats presented sexually dichotomous impairments characterised by early autistic-like traits in males – but not in females – associated with forebrain white matter tract alterations^32^. This was also shown in other rodent models of maternal immune activation triggered by other pathogen-associated molecular patterns, namely lipopolysaccharide from *E*. *coli* and Poly(I:C) mimicking a viral infection^50–54^. Male – but not female – fetuses presented increased blood levels of IL-1β, and fetuses from both sexes *in-utero* exposed to GBS had increased blood levels of TNF-α. Sex-specific decreased myelination, decreased white matter thickness, microglial depletion in the corpus callosum and neuronal apoptosis has been reported in this rat model, as well as other rodent models of maternal GBS-induced inflammation^32–34,55^. IL-1 holds an important function in neuroimmune responses, including a role in the induction of neuronal death and altered myelination^56,57^. Following their release from the placenta and circulation in the fetal bloodstream, as observed in our results, IL-1β and TNF-α have been shown by others to cross the blood-brain barrier and induce neurotoxicity through elevating the glutamate production, inducing neuronal necrosis, or through activation of neurono-glial necroptosis or apoptosis^58,59^. TNF-α can induce cell death in mature oligodendrocytes and apoptosis in developing oligodendrocytes, potentially leading to reduced myelination and diffuse white matter injuries^60,61^. The sex-specific placental and fetal pro-inflammatory responses might be relevant to the higher susceptibility of the male population for preterm birth, brain injuries and neurodevelopmental disorders. Acute placental inflammation has been associated with a 3-fold increased risk of autism spectrum disorders, with males being more susceptible than females^62^.

While the regulatory cytokine IL-10 does not seem to play a role other than a normal homeostatic response, the observed difference in IL-1β levels between males and females might be the consequence of sex-specific IL-1Ra levels, that better protect females against inflammation. Clinical studies have shown that higher concentrations of IL-1Ra are present in the amniotic fluid of females compared to males, and some authors suggest that maternal decidua secretes higher levels of IL-1Ra in the presence of a female fetus^63–65^. These studies are in line with the decreased IL-1Ra in males compared to females, and the increased IL-1β staining intensity in the decidual compartment associated with males *versus* females exposed to GBS. Other preclinical and clinical studies have shown in various infectious/inflammatory contexts a higher IL-1Ra and lower IL-1β release in females, and the opposite findings in males^53,66,67^. These results could be due to the influence of sex hormones or to sex-differentiated segregation of genetic polymorphisms that increase the production of IL-1Ra which are more frequent in women than men, or due to other mechanisms^68,69^. Sex hormones might influence the production of IL-1 by immune cells. The androgen receptor (AR) is expressed on innate immune cells, including male and female PMNs, which are the main inflammatory cells involved in chorioamnionitis^70–73^. Studies using conditional AR knock-out showed that AR signalling up-regulates the innate immune response^71^. In keeping with the GBS-induced up-regulation of pro-inflammatory cytokine response in males as found in our model, it is known that male rat fetuses produce a peak of masculinising testosterone between G19 and P4. This time frame overlaps with the timing of the highest incidence of chorioamnionitis in humans (third tier of gestation) and with the timing of immune stimulation in our model^74^. Hence, sex-specific GBS-induced placental and fetal IL-1β response might result from a stronger pro-inflammatory reaction driven by androgen in males.

In summary, our results are shedding further insight into the inflammatory mechanisms that lead to the sex-skewed ratio of neurobehavioural effects following a placental infection. Additional insights into the mechanisms underpinning the pathophysiology of sex-differentiated pathogen-induced placental injuries are needed to develop personalised therapeutic interventions, to better control the infectious and inflammatory components of chorioamnionitis.

## Methods

### Bacterial strains and growth conditions

A stock of β-hemolytic capsular serotype Ia GBS strain (strain #16955) stored at −80 °C in brain heart infusion (BHI) broth with 15% glycerol was used for all experiments^32^. Bacterial preparation was executed as described^32^. Briefly, on gestational day (G) 18, GBS was inoculated into sterile brain heart infusion (BHI) broth and incubated with shaking (240 rotation per minute [rpm]) for 18 h at 37 °C. At G19 AM, this GBS culture was re-inoculated into sterile BHI at initial absorbance 0.05 (optical density [OD]_600 nm_) and incubated at 37 °C while shaking until bacteria reached their exponential growth phase, *i*.*e*. when absorbance reached 0.70 (OD_600 nm_). A volume of 20 ml was centrifuged and precipitated bacteria were washed twice in 20 ml of sterile 0.9% saline. The precipitate was resuspended in 2 ml of sterile 0.9% saline, obtaining a final dose of 10^8^ colony forming units per 100 µl. The aliquot was kept on ice before infections. Serial dilutions from 10^−5^ to 10^−10^ were plated in triplicate on BHI agar and incubated overnight at 37 °C. Bacterial counts were determined by counting colonies per 100 µl spread on the agar plates, to confirm the exact infection dose. To control for contamination, samples from β-hemolytic GBS doses were plated in duplicate on Columbia blood agar 5% with sheep blood medium (Thermo Scientific, MA, US) and on CHROMID Strepto B agar (BioMérieux, Saint-Laurent, QC, Canada), a selective chromogenic medium for the screening of GBS.

### *In-vivo* experimental model of end-gestational GBS infection and ethics statement

All procedures were approved by the Research Institute of McGill University Health Centre (RI-MUHC) Animal Care Committee (protocol #2015-7675). All experiments were executed in accordance with the Canadian Council on Animal Care guidelines. Thirty pregnant primiparous Lewis rats obtained from Charles River Laboratories (Kingston, NY, US) arrived in four different cohorts at the animal facility on gestational day (G) 13. The animals were kept in a clean pathogen-free facility at the RI-MUHC (Glen site, Montreal, QC, Canada), in a controlled environment (12-h light/dark cycle) with water and food *ad libitum*. Cohorts of dams were injected intraperitoneally at gestational day (G) 19 with either (group 1) 100 µl of sterile 0.9% saline (control [CTL], number [n] of dams = 13) or (group 2) β-hemolytic serotype Ia GBS suspended in 100 µl of sterile 0.9% saline (GBS group, n = 17). Injections were done every 1 h, starting from 10 AM, to avoid a time effect between inoculated dams. The exact concentrations of the injected doses of β-hemolytic GBS serotype Ia were between 8.9 × 10^7^ CFU/100 µl and 3.7 × 10^8^ CFU/100 µl.

### Gestation monitoring and Cesarean (C)-sections

Dams were weighed daily and monitored twice a day to detect any sickness behaviour. Dams underwent C-sections from 10 AM, with respect to their injection time. C-sections were performed in four separated cohorts to reduce the time between the first and the last inoculated dam within the experiment. C-sections were performed under general anesthesia (isoflurane 2%) at 24 h (n = 3 CTL and n = 4 GBS), 48 h (n = 5 CTL, n = 5 GBS) and 72 h (n = 5 CTL, n = 8 GBS) to collect maternofetal tissues with respect to fetal sex. Following the injection of a single-dose of β-hemolytic GBS serotype Ia on G19, four out of five (80%) and six out of eight (75%) dams presented GBS infiltrates in their placentas at, respectively, 48 h and 72 h. Dams, placentas associated with alive fetuses displaying these placental GBS infiltrates were included in the present study. Only the placentas from alive fetuses were included in the analyses.

### Tissue sampling and processing

All fetuses were taken along with their placenta while dams remained under deep anaesthesia. The position of each fetus in the uterine horn was noted to evaluate whether the position influenced GBS infection and mortality. No differences were seen due to the implantation site. Blood from all alive fetuses was collected with Lithium Heparin Gel Separator tubes (BD Microtainer blood collection tubes, BD, NJ, US) following decapitation. Tubes containing blood samples were then centrifuged and aliquoted. Fetal serum samples were kept at −80 °C until analysis. The number of dead fetuses was determined to evaluate the fetal mortality rate per litter. Fetal tails were collected to determine the sex of fetuses by amplification of a sequence within the SRY gene (Y chromosome) using polymerase chain reaction (PCR), as described^37^. Placentas were separated from the fetuses, and cut at the median coronal section (*i*.*e*. at the level of the umbilical cord): one half was fast-frozen (covered jar containing 2-methylbutane on dry ice) and kept at −80 °C until analysis and the adjacent section, including the umbilical cord and membranes, was fixed in 4% buffered formaldehyde, processed, and paraffin-embedded. Following placental and fetal tissue sampling, maternal blood from the abdominal aorta was collected with Lithium Heparin Gel Separator tubes (BD, NJ, US), centrifuged, aliquoted and stored at −80 °C until analysis.

### Histopathology examination and immunohistochemistry

Two subsequent 5-µm thick sections of paraffin-embedded placentas were used per slide for *in-situ* analysis. GBS serotype Ia, polymorphonuclear cells (PMNs) and interleukin (IL)−1β staining were investigated by immunohistochemistry. Slides were dewaxed (5 × 5 min in xylene) and hydrated, peroxidase was quenched (3% H_2_O_2_ in methanol, 0.5%) and antigen retrieval was performed by incubating slides in boiling sodium citrate buffer solution (pH 6.0) for 20 min in a pressure cooker. Slides were washed three times in TBS and incubated in a humid chamber at room temperature for 2 h with primary antibodies. GBS serotype Ia infiltrates were determined using a rabbit GBS serotype Ia group-B typing antisera set (270023 Ia, 1:500; Denka Seiken Co., Tokyo, Japan). Rabbit anti-PMN antibody (CLA51140, 1:100; Cedarlane Lab, ON, Canada) was used to detect PMN infiltrates throughout placental compartments (decidua, junctional zone, labyrinth and amnion), a key marker for histologic chorioamnionitis. Rabbit anti Rat interleukin (IL)-1β (AAR15G, 1:100, Bio-Rad, CA, US) was used to measure IL-1β staining intensity with respect to the placental compartment. Sections were washed three times with TBS following primary antibody incubation, and slides were incubated with secondary antibodies in a humid chamber at room temperature for 45 min. Mouse horseradish peroxidase (HRP)-conjugated anti-rabbit (sc-2357, 1:100, Santa Cruz Biotechnology, TX, US) was used as the secondary antibody. The diaminobenzidine molecule (DAB) was used to produce a precipitate for immunohistochemical quantification. Slides were counterstained with hematoxylin. Additional sets of placental sections treated similarly but without the primary antibody were used as negative controls.

### Image analysis and quantification

The slides were scanned with a NanoZoomer Digital Pathology (NDP) Scanner (NanoZoomer 2.0-RS, Hamamatsu Photonics). Densities of PMNs in different placental compartments were determined as previously described^32^. Briefly, the number of PMNs was counted in five pre-determined fields in the labyrinth, five fields in the junctional zone, three fields in the decidua, and two fields in the amnion. Densities were calculated with the number of counted cells divided by the field area. The mean density per anatomical compartment was used in the statistical analysis. Immunohistochemistry staining for IL-1β was quantified using the IHC profiler methodology outlined previously^75^. The percentage of positive high staining intensity for IL-1β was used in the analysis. All the analyses were done by an investigator blinded to the experimental conditions.

### Placental protein extraction, determination and quantification

Total proteins were extracted from placental tissues using the BCA Protein Assay Kit (Thermo Scientific-Pierce). Whole protein isolates were quantified using Bradford Protein Assay (Bio-Rad, ON, Canada). Quantified protein isolates of whole placental tissues were aliquoted in samples of 120 µl and stored at −80 °C until analysis. Key pro-inflammatory cytokines (IL-1β [RLB00, R&D Systems, MN, US], IL-18 [ELR-IL18, RayBiotech, GA, US], IL-6 [R6000B, R&D Systems, MN, US] and TNF-α [RTA00, R&D Systems, MN, US]), IL-1 receptor antagonist (IL-1Ra [RI0322, Neo Scientific, MA, US]), GBS-associated immunosuppressive cytokine IL-10 (R1000, R&D Systems, MN, US), PMN-chemoattractant CXCL1/CINC-1 (RCN100, R&D Systems, MN, US), PMN-associated antimicrobial protein S100A9 (LS-F18545, Lifespan Biosciences, WA, US) and the PMN-produced MMP-8 (ELR-MMP8, RayBiotech, GA, US) were measured by selective enzyme-linked immunosorbent assays (ELISA) in protein extracts from the same placentas. ELISA results for placentas were calculated with the protein concentration per sample, previously measured by Bradford. Before ELISAs, all serum aliquots from female and male fetuses within the same litter were pooled with respect to sex. Serum and placenta samples from the same timepoint and both sexes from the same litter were assessed on the same ELISA plate to control for inter-experiment variability. Samples were measured in duplicate, and the mean of the replicates was used in the statistical analysis. ELISAs were carried out as per manufacturer’s instructions.

### Statistical analysis

As treatments were administered prenatally and each pup belongs to one specific treated dam and to avoid the artificial sample size inflation caused by treating each rat from the same litter as an independent number (n), one male and one female per treated dam were used as n = 1 per litter. Statistical analyses were performed using the statistical package IBM Statistics 25 (SPSS). Assessments of normality of distribution and homogeneity of variance for each data set were initially conducted by a Shapiro-Wilk normality test and Levene’s test (p > 0.05), respectively. All data related to placentas and fetuses were analysed using linear mixed models (MIXED, SPSS), using *Sex* (Female, Male) and *Treatment* (CTL, GBS) as fixed effects and *LitterID* as a random effect. Including the litter identification in our statistical design allows within-litter between-sex comparisons, *i*.*e*. inflammatory responses of male and female pups from the same dam. Sidak pairwise comparisons were applied in SPSS when there was a significant interaction between *Sex* and *Treatment* at the level of p < 0.05. The data sets for each cytokine tittered in maternal sera were analysed by unpaired two-tailed Mann-Whitney U tests or unpaired two-tailed Student’s T tests when data were, respectively, not normally or normally distributed. Two-way Analysis of Variance (ANOVA, general linear model in SPSS) was performed to analyse the mean weight of dams between treatments (between-subjects; CTL, GBS) at three gestational timepoints (within-subject repeated measures; G20, G21, and G22). Correlations between data sets were analysed by linear regression and the goodness of fit was calculated using Graph Pad Prism software version 8.00 for Windows (Graph Pad Software, San Diego, CA). All figures were constructed using Graph Pad Prism 8.00. Data are presented as the mean ± standard error of the mean (SEM).

## Data Availability

The data sets used for the current study are available from the corresponding authors upon reasonable request.
